# Fatigue and physical activity in cancer survivors: A cross‐sectional population‐based study

**DOI:** 10.1002/cam4.2060

**Published:** 2019-03-12

**Authors:** Margarida Matias, Giulia Baciarello, Mohamed Neji, Antonio Di Meglio, Stefan Michiels, Ann H. Partridge, Marc Karim Bendiane, Karim Fizazi, Michel Ducreux, Fabrice Andre, Ines Vaz‐Luis

**Affiliations:** ^1^ Department of Medical Oncology Gustave Roussy, Université Paris‐Sud, Université Paris‐Saclay Villejuif France; ^2^ Departement of Medical Oncology Centre Hospitalier Intercommunal de Créteil Créteil France; ^3^ INSERM UMR 981 Gustave Roussy Villejuif France; ^4^ Department of Biostatistics and Epidemiology Gustave Roussy, Université Paris‐Sud, University Paris‐Saclay Villejuif France; ^5^ Department of Medical Oncology, Dana‐Farber Cancer Institute Boston Massachusetts; ^6^ Provence‐Alpes‐Côte d'Azur Regional Health Observatory, INSERM UMR912 Marseille France

**Keywords:** cancer, fatigue, physical activity, quality of life, survivorship

## Abstract

**Purpose:**

A substantial proportion of cancer survivors experience fatigue after diagnosis. Physical activity (PA) can impact fatigue after cancer. In this study, we evaluated the prevalence and association of fatigue and the practice of PA in a population with early cancer.

**Methods:**

Using the national population‐based French cross‐sectional study *Vie après le cancer 2*, we included 1984 patients with early breast (61.1%), prostate (21.5%), and colorectal (17.4%) cancer. Severe fatigue at 2 years postdiagnosis was defined by a score ≥40 in the European Organization for Research and Treatment of Cancer quality of life questionnaire (EORTC QLQ C30) fatigue subscale. PA was defined as (a) self‐reported PA before diagnosis (active/inactive) and (b) change in PA since diagnosis (increased/maintained exposure vs decreased exposure/remaining inactive). Multivariate regression examined associations of severe fatigue with PA, adjusting for baseline clinical and treatment variables.

**Results:**

Median age was 52 years. 51.5% of patients experienced severe fatigue 2 years post‐diagnosis. 87.7% reported to be physically active before cancer diagnosis; 53.3% of patients either decreased PA or remained inactive at 2 years postdiagnosis. At 2 years postdiagnosis, severe fatigue was associated with a change in PA since diagnosis: patients with decreasing PA/remaining inactive from pre‐ to postdiagnosis had a higher risk of severe fatigue vs those with increasing/maintaining PA (adjusted odds ratio [95% confidence interval] 2.32 [1.85‐2.90]).

**Conclusion:**

Fatigue continues to be a substantial problem for cancer survivors 2 years after cancer diagnosis and is associated with PA decreasing/remaining inactive since diagnosis. Interventions to maintain or increase PA for cancer survivors should be tested to mitigate long‐term fatigue after cancer.

## 1 INTRODUCTION

There are currently more than 24 million cancer survivors between the United States and Europe. Prostate, breast, and colon cancer represent the most prevalent cancers and the vast majority of these patients will be alive at 5 years after diagnosis^1^, ^2^; in Europe, the 5‐year relative survival rate is ≈80.0% for prostate and breast cancer and ≈60.0% for colon cancer.^3^

Nevertheless, a substantial proportion of these patients will experience long‐term toxicity associated with cancer treatments that can have a substantial impact on comorbidities, quantity and quality of life, social and psychological functioning.^4^, ^5^ Therefore, management of long‐term side effects of cancer treatments has become an essential part of clinical cancer care for this growing group of cancer survivors.

Cancer‐related fatigue represents one of the most frequent and distressing side effects of cancer treatment.^6^ Based on prior studies, most of which have small sample sizes, cancer related fatigue appears to be related to several pretreatment characteristics including: (a) *contextual characteristics* such as marital status and low income; (b) *patient characteristics* such as younger age, presence of comorbidities pre‐existing fatigue, high body mass index (BMI), and concomitant complaints of loneliness, high levels of stress, depression, anxiety, and sleep disturbances^6^, ^7^; (c) *tumor characteristics* such as advanced tumor stage; and (d) *treatment characteristics* such as intensity and type of treatment.^8^, ^9^ Previous research, including a meta‐analysis,^10^ has also suggested that behavioral interventions such as increasing physical activity (PA) may have a favorable impact on the modulation of fatigue^11^, ^12^, ^13^, ^14^, ^15^ which led to the general recommendation of proposing exercise for those who experience fatigue after cancer.^16^

*Vie après le cancer 2* (VICAN 2) study is a multi‐institutional national French study, in which patients were surveyed 2 years after diagnosis. Patient‐reported outcomes included fatigue determination and ad hoc self‐assessment questions focused on PA. In this study, we used VICAN 2 to describe, at 2 years post cancer diagnosis, the prevalence of fatigue, the exposure to PA practice, and their associations. The purpose of this analysis is to investigate the magnitude of the problem of long‐term fatigue among French cancer survivors and the use of PA among this population.

## 2 METHODS

### 2.1 Study data source and cohort

#### 2.1.1 Data source

VICAN 2 is a multi‐institutional national French cross‐sectional study including patients between 18 and 82 years of age, who were diagnosed with a first cancer between January and June 2010, and who were enrolled in one of the main French Health Insurance programs at that time (Schemes Caisse Nationale de l' Assurance Maladie des Travailleurs Salariés, Régime Social des Indépendants, and Mutuelle Sociale Agricole). Together, these three programs covered over 90.0% of the French population.

VICAN 2 includes cancers of 12 tumor sites that account for 88.0% of cancers in France (breast, colorectal, prostate, lung, melanoma, head and neck, bladder, kidney, thyroid, cervix, endometrial, and non‐Hodgkin lymphoma) and is restricted to French‐speaking patients who were living in France for at least 2 years at the time of inclusion.^15^ The final analytic cohort for VICAN 2 encompasses 4347 patients. Study data were collected from via telephone interviews with each individual patient 2 years after diagnosis. Patients were required to answer questionnaires covering several domains including: sociodemographic, circumstances of diagnosis, relationships with healthcare providers and healthcare system, treatment received and perceived side effects, diet and PA behavior, alcohol and tobacco consumption, and fatigue. Interviewers used an already standardized and validated set of questionnaires (the European organization for research and treatment of cancer quality of life questionnaire [EORTC]‐QLQ C30),^17^ available on the “*Institut National du Cancer*” (INCa) website.^18^

A medical questionnaire concerning each patient was also collected by the physician who had initiated cancer treatment, compiling the following information: tumor histology, grade, size, stage, and type of treatment received. The medical survey was completed by 87.7% of participants^15^ and is available on the “*Centre Maurice Halbwachs*” web site.^19^ Additional details on the VICAN 2 study have been previously published.^15^

#### 2.1.2 Study cohort

Of the 4347 patients enrolled in VICAN 2, we selected only patients with breast, prostate and colorectal cancer (n = 2315), since these are the most prevalent diagnoses among long‐term cancer survivors being therefore good survivorship models. Furthermore, we excluded patients with evidence of distant and local relapse (n = 204), patients with a second malignancy (n = 23), patients who experienced serious events (eg, cerebral stroke, car accidents [n = 64]) between diagnosis and the time of survey (2 years after diagnosis) and patients under treatment by chemotherapy at the time of survey (n = 34). There was a 99.7% completion rate of all fatigue‐related questions. Patients that did not answer at least 2 out of 3 fatigue‐related questions from the EORTC QLQ C30 questionnaire were also excluded (n = 6).

**Figure 1:**
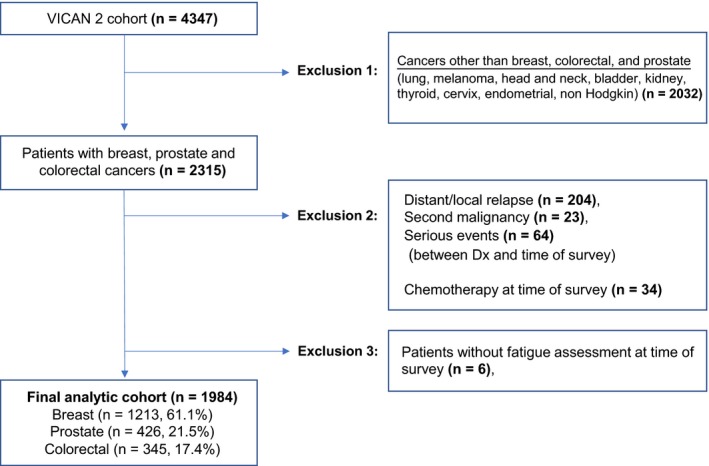
Flow diagram of the patient population. VICAN: *Vie après le cancer*; Dx: diagnosis

The final analytic cohort included 1984 patients (Figure 1).

### 2.2 Key variables

#### 2.2.1 Primary outcome

The primary outcome of interest was the presence of severe fatigue at 2 years after diagnosis, defined by a score of EORTC QLQ‐C30 ≥ 40.^20^, ^21^, ^22^, ^23^, ^24^

#### 2.2.2 Independent variables

Our main independent variable was PA defined (a) practice of PA before diagnosis (active vs inactive), (b) change in PA since diagnosis (decreased exposure/remaining inactive vs increased/maintained exposure). These two variables were assessed using the following questions: “Before your illness, did you practice regular PA (sport, gardening, household, walking, in the context of your work/hobbies? (yes, every day; yes, less often; no) and “Since the diagnosis of your illness, have you modified your PA? (yes, more than before; yes, less than before; yes, completely stopped; no).

#### 2.2.3 Covariates

We also looked at two main groups of variables: (a) Patient, social, demographic, and tumor features and (b) treatment features. The patient, social demographic and tumor features included: gender (female, male), age at diagnosis (continuous variable), marital status (in couple, alone), educational level (high school or more, less than high school) and employment (employed/formation, unemployed, retired/invalidity), comorbidities (no, yes), pain in the last 15 days (never, sometimes, often, very often, constantly), BMI at diagnosis (underweight [<18.5], normal [18.5‐24.9], overweight [25.0‐29.9], obese [≥30.0]), BMI variation since diagnosis (−10 to +10%, ≤‐10%, ≥+10%), alcohol consumption (<4/week; ≥4/week), smoking (no, yes). In addition, among smokers, the following information was also collected for a descriptive purpose: smoking frequency (daily, less than daily), numbers of cigarettes per day (1‐5, 6‐10, 11‐20, more than 20, other). The treatment features included: surgery (no, yes), chemotherapy (no, yes), radiation therapy (no, yes), and endocrine therapy (no, yes).

### 2.3 Statistical analysis

First, we performed a baseline description of our cohort. We examined the presence of severe fatigue by the prespecified independent variables and covariates, using chi‐square test for categorical variables and analyses of variance for continuous variables. Secondly, we used multivariate logistic regression models to evaluate the association of severe fatigue with the pre‐specified independent variables and covariates. Patients were stratified by tumor site (breast vs prostate vs colorectal cancer). A complete dataset analysis was performed. Sensitivity analyses using linear regression models with fatigue modeled as a continuous variable were also performed. All statistical analyses were performed with Stata software version 14.0. Two‐sided *P* values less than 0.05 were considered statistical significant.

## 3 RESULTS

### 3.1 Characteristics of the overall study population

Median age at diagnosis was 52.0 years (interquartile range [IQR]: 45‐65). The vast majority were breast cancer survivors (61.1%), and 21.5% and 17.4% were prostate and colorectal cancer survivors, respectively. Nighty‐one percent of patients underwent surgery, 45.4% received chemotherapy and 64.2% received radiation therapy. Forty‐six percent of patients had received or were still on endocrine therapy (Table 1).

**Table 1:** Characteristics of the whole cohort and by report of severe fatigue at 2 years after diagnosis

Characteristics	Whole cohort N (%)		No severe fatigue N (%)	Severe fatigue N *(%)	Adjusted OR, (95%CI)
Overall	1984 (100)		962 (48.5)	1022 (51.5)	—
PA before Dx					
Active	1740 (87.7)		849 (48.8)	891 (51.2)	1
Inactive	231 (11.6)		102 (44.2)	129 (55.8)	0.92 (0.66‐1.27)
Missing	13 (0.7)		11 (84.6)	2 (15.4)	—
Change in PA since Dx					
Increased/maintained PA	911 (45.9)		557 (61.1)	354 (38.9)	1
Decreased PA/remained inactive	1058 (53.3)		393 (37.2)	665 (62.9)	2.32 (1.85‐2.90)*
Missing	15 (0.8)		12 (80.0)	3 (20.0)	—
Tumor site					
Breast	1213 (61.1)		452 (37.3)	761 (62.7)	1
Prostate	426 (21.5)		312 (73.2)	114 (26.8)	0.997 (0.51‐1.95)
Colorectal	345 (17.4)		198 (57.4)	147 (42.6)	1.17 (0.73‐1.87)
Gender					
Female	1374 (69.3)		531 (38.7)	843 (61.4)	1
Male	610 (30.8)		431 (70.7)	179 (29.3)	0.63 (0.38‐1.04)
Age at diagnosis					
Median (IQR)	52 (45‐65)		60 (48‐68)	49 (44‐60)	0.97 (0.95‐0.98)*
Marital status					
In couple	16 01 (80.7)		783 (48.9)	818 (51.1)	1
Not in a couple	365 (18.4)		165 (45.2)	200 (54.8)	0.93 (0.70‐1.23)
Missing	18 (0.9)		14 (77.8)	4 (22.2)	—
Education					
High school diploma or higher	1390 (70.1)		653 (47.0)	737 (53.0)	1
Less than high school	588 (29.6)		306 (52.0)	282 (48.0)	1.07 (0.82‐1.38)
	6 (0.3)		3 (50.0)	3 (50.0)	—
Employment					
Employed/formation	887 (44.7)		369 (41.6)	518 (58.4)	1
Unemployed	298 (15.0)		90 (30.2)	208 (69.8)	1.01 (0.72‐1.40)
Retired/unable to work	790 (39.8)		494 (62.5)	296 (37.5)	0.95 (0.64‐1.41)
	9 (0.45)		9 (100.0)	0 (0)	—
Comorbidities					
No	1059 (53.4)		559 (52.8)	500 (47.2)	1
Yes	925 (46.6)		403 (43.6)	522 (56.4)	1.68 (1.34‐2.11)*
Pain in the last 15 days before survey					
Never	603 (30.4)		459 (76.1)	144 (23.9)	1
Sometimes	644 (32.5)		326 (50.6)	318 (49.4)	2.14 (1.63‐2.80)*
Often	341 (17.2)		91 (26.7)	250 (73.3)	5.37 (3.85‐7.51)*
Very often	255 (12.9)		58 (22.8)	197 (77.3)	6.30 (4.30‐9.21)*
Constantly	139 (7.0)		27 (19.4)	112 (80.6)	7.39 (4.47‐12.2)*
Missing	2 (0.1)		1 (50.0)	1 (50.0)	—
BMI before Dx					
Underweight (<18.5)	43 (2.2)		13 (30.2)	30 (69.8)	1.42 (0.67‐3.01)
Normal (18.5‐24.9)	1056 (53.2)		473 (44.8)	583 (55.2)	1
Overweight (25.0‐29.9)	630 (31.8)		359 (57.0)	271 (43.0)	0.80 (0.62‐1.04)
Obese≥30.0)	243 (12.3)		115 (47.3)	128 (52.7)	0.90 (0.64‐1.28)
Missing	12 (0.6)		2 (16.7)	10 (83.3)	—
BMI variation since Dx					
>‐10% and <+10%		1652 (83.3)	868 (52.5)	784 (47.5)	1
≤‐10.0%		78 (3.9)	23 (29.5)	55 (70.5)	2.01 (1.14‐3.55)**
≥+10.0%		238 (12.0)	68 (28.6)	170 (71.4)	1.22 (0.86‐1.73)
Missing		16 (0.8)	3 (18.8)	13 (81.3)	—
Alcohol consumption					
<4/week	1559 (78.6)		699 (44.8)	860 (55.2)	1
≥4/week	419 (21.1)		257 (61.3)	162 (38.7)	0.99 (0.75‐1.33)
Missing	6 (0.3)		6 (100.0)	0 (0)	—
Smoking					
No	1623 (81.8)		814 (50.2)	809 (49.9)	1
Yes	356 (17.9)		143 (40.2)	213 (59.8)	0.997 (0.75‐1.33)
Missing	5 (0.3)		5 (100)	0 (0)	—
Surgery					
No	169 (8.5)		109 (64.5)	60 (35.5)	1
Yes	1814 (91.4)		852 (47.0)	962 (53.0)	0.63 (0.40‐1.002)
Missing	1 (0.1)		1 (100)	0 (0)	—
Chemotherapy					
No	1084 (54.6)		628 (57.9)	456 (42.1)	1
Yes	900 (45.4)		334 (37.1)	566 (62.9)	1.22 (0.95‐1.56)
Radiation therapy					
No	708 (35.7)		455 (64.3)	253 (35.7)	1
Yes	1273 (64.2)		506 (39.8)	767 (60.3)	1.38 (1.02‐1.88)***
Missing	3 (0.2)		1 (33.3)	2 (66.7)	
Endocrine therapy					
No	1060 (53.4)		607 (57.3)	453 (42.7)	1
Yes	921 (46.4)		353 (38.3)	568 (61.7)	1.13 (0.87‐1.48)
Missing	3 (0.2)		2 (66.7)	1 (33.3)	—

PA: physical activity; Dx: diagnosis

*P < 0.001; ** *P* = 0.016; *** *P* = 0.036

Most of patients (87.7%) reported having been physically active before the diagnosis. Among the whole cohort, 45.9% increased or at least maintained PA after diagnosis, whereas 53.3% reported reducing, quitting, or never engaging in PA after cancer diagnosis. Table 2 represents other health behaviors that were reported in this population.

**Table 2:** Descriptive of other health behaviors characteristics

Overall population	N (%) 1984 (100.0)
BMI before Dx	
Underweight (<18.5)	43 (2.2)
Normal (18.5‐24.9)	1056 (53.2)
Overweight (25.0‐29.9)	630 (31.8)
Obesity (≥30.0)	243 (12.3)
Missing	12 (0.6)
BMI variation since diagnosis	
>‐10% and <+10%	1652 (83.3)
≤‐10.0%	78 (3.9)
≥+10.0%	238 (12.0)
Missing	16 (0.8)
Smoking	
No	1623 (81.8)
Yes	356 (17.9)
Missing	5 (0.3)
Smoking frequency	
Non smokers	1623 (81.8)
Every day	259 (13.1)
Less often	97 (4.9)
Missing	5 (0.3)
Number of cigarettes	
Not concerned	1623 (81.8)
1‐5	62 (3.1)
6‐10	87 (4.4)
11‐20	88 (4.4)
Over 20	19 (1.0)
Other	3 (0.2)
Missing	102 (5.1)
Alcohol consumption	
Never	422 (21.3)
≤1/month	336 (16.9)
2‐4/month	515 (26.0)
2‐3/week	286 (14.4)
≥4/week	419 (21.1)
Missing	6 (0.3)

BMI: Body mass index; Dx: diagnosis.

Table S1 describes PA by tumor site (breast, prostate, and colorectal cancer).

### 3.2 Prevalence of severe fatigue after diagnosis and associations with patients characteristics

**Figure 2:**
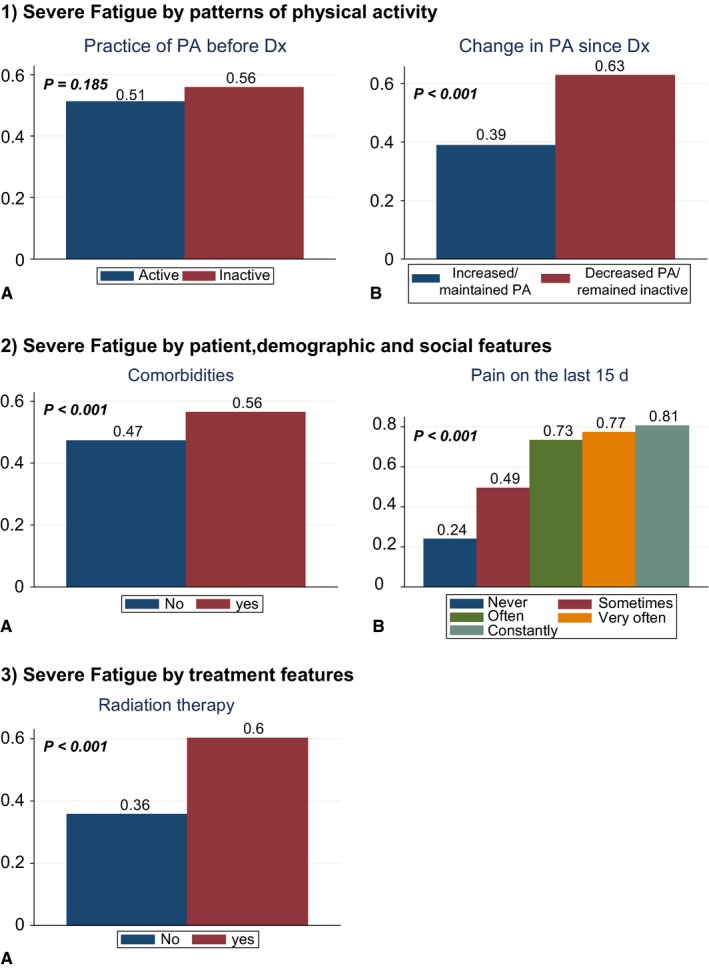
Univariate associations of severe fatigue by: (1) Patterns of physical activity—(A) before diagnosis, (B) by change in physical activity since diagnosis (2) Patient, demographic, and social features—(A) comorbidities, (B) pain on the last 15 days (3) Treatment features—(A) radiation therapy. Note: *p*: *P*‐value (chi squared test). PA: physical activity, Dx: diagnosis

Fifty‐two percent of patients reported severe fatigue 2 years after diagnosis. Severe fatigue was associated with PA and several other patient, demographic, social, and treatment features in both univariate and multivariate analyses. (Table 1 and Figure 2).

#### 3.2.1 Physical activity

There was no impact of exposure to PA before diagnosis on prevalence of severe fatigue after 2 years of diagnosis (inactive vs active patients before diagnosis: severe fatigue 55.8% vs 51.2, unadjusted *P* = 0.185; adjusted Odds Ratio [aOR], 95% confidence interval [95%CI] 0.92, 0.66‐1.27). Nevertheless, we observed a higher rate of severe fatigue among patients who decreased PA/remained inactive after diagnosis when compared to patients who increased/maintained PA after cancer (62.9% vs 38.9%; aOR [95%CI] 2.32, 1.85‐2.90).

#### 3.2.2 Patient, demographic, social features

The following other cohort characteristics were associated with severe fatigue: age (decreasing risk of severe fatigue with increasing age (aOR, [95%CI] for *age as a continuous variable*=0.97, [0.95‐0.98]); comorbidities, (56.4% of severe fatigue among patients with comorbidities vs 47.2% among those without comorbidities (aOR, [95%CI] =1.68, [1.34‐2.11]); and pain (prevalence of severe fatigue increased according to increasing frequency of reported pain: specifically, severe fatigue was present among 49.4% of patients who sometimes had pain, 73.3% of those who often had pain, 77.3% of those who had pain very often and 80.6% of those who constantly had pain vs 23.9% of those who never reported pain, respective aORs [95%CI] *vs never reporting pain* 2.14, [1.63‐2.80]; 5.37, [3.85‐7.51]; 6.30, [4.30‐9.21] and 7.39, [4.47‐12.2]).

#### 3.2.3 Treatment features

A higher rate of severe fatigue was also reported among patients who had received radiation therapy (60.3% vs 35.7 aOR, [95%CI] yes vs no = 1.38, [1.02‐1.88]). Most of the patients performed surgery (91.4%). There was a trend towards reduction of severe fatigue among patients treated with surgery vs those not treated with surgery (aOR [95%CI]: 0.63 [0.40‐1.002]).

Results from sensitivity analyses were consistent with the main analysis (data not shown).

## 4 DISCUSSION

In a large, contemporary, population‐based cohort of 1984 French breast, prostate and colorectal cancer survivors, we found that the vast majority of patients (51.5%) reported severe fatigue 2 years after cancer diagnosis. Although over 90% of patients in this cohort reported having been physically active before diagnosis, a substantial proportion (53.3%) of patients stopped, decreased PA or remained inactive after cancer diagnosis. When examining associations with severe fatigue, this was linked with several demographic, social and treatment features, but also with health behaviors such as change in PA after diagnosis.

Among patients with early stage cancers, most of prior studies consistently have suggested that severe fatigue after cancer treatment is a relevant issue among a considerable population of cancer patients and is associated with significant functional and social impact. Indeed, there are several observational studies reporting a wide range spanning from ≈30 to up to 70% of patients that may suffer from severe fatigue over their survivorship period.^4^, ^24^, ^25^, ^26^, ^27^ The heterogeneity of reported severe fatigue prevalence is probably due to several factors including differences in the definition of severe fatigue, type of measures used, diversity in populations under study and follow‐up times. To better explore this, we used a standardized and validated scale for the evaluation of fatigue (EORTC QLQ C30 fatigue subscale) and a large population‐based cohort, allowing us to confirm that severe fatigue is a substantial clinical problem among at least half of the most prevalent French cancer survivor populations.

Despite the prevalence of this symptom, limited available research suggests that fatigue is markedly underreported by patients and therefore also under‐treated.^9^ The complexity and multidimensional features of this symptom may partially explain this relevant gap in addressing such an important issue in survivorship care.

Our findings shed further light on the relationship between prevalence of long‐term severe fatigue and exposure to PA pre and post cancer diagnosis. Two main aspects in this relationship are noteworthy. First, despite a very high proportion of patients reporting to engage to any extent in PA before diagnosis (almost 90%), there seems to be no association of pre‐diagnosis PA and prevalence of long‐term fatigue among cancer survivors in our population. Second, in our cohort increasing or maintaining the same level of pre‐diagnosis exposure to PA after cancer diagnosis was significantly associated with a reduced risk of severe fatigue 2 years after diagnosis, a finding that is in line with prior studies showing that PA can positively impact fatigue, including a recent meta‐analysis of 39 studies which found an improvement of cancer related fatigue with increased PA, (weighted effect size 0.33 [95% CI, 0.24‐0.43]).^10^ Interestingly, there is a growing body of evidence suggesting that chronic inflammation through neuro‐immune activation might be a central contributor to posttreatment fatigue^28^, ^29^, ^30^, ^31^, ^32^ and it is therefore possible that PA exerts its role on mitigating fatigue through the modulation of the inflammation pathways.^33^, ^34^, ^35^

Taken together, these results indicate that there is room to promote PA practices in this setting, specifically targeting interventions aimed at modulating severe fatigue among the large proportion of patients (53.3%) that reports to have stopped, decreased, or continued to stay inactive after their cancer diagnosis. The issue of sedentary behavior and of the reduction in PA exposure following the diagnosis of cancer is well described also among different populations: for example, among a population of cancer patients in the United States, only 37.0% of breast, 43.0% of prostate, and 35.0% of colorectal cancer survivors would engage in PA as recommended by guidelines.^36^

Concordant with other studies, our analysis also suggests that younger age at diagnosis, the presence of comorbidities, high frequency of pain and treatment (type o surgery and receipt of radiotherapy) are associated with fatigue.^6^, ^7^, ^8^, ^9^, ^37^, ^38^, ^39^, ^40^ In particular, previous studies had shown that younger people have more fatigue than older people.^41^, ^42^ In addition, the association of fatigue and pain is well established, and many authors have investigated the etiology and temporal characteristics of their relationship. In our study, a higher frequency of reported pain corresponded to increasingly higher odds of severe fatigue. As previously suggested radiotherapy use was associated with fatigue.^43^, ^44^ Concerning surgery, although a numerically higher percentage of patients who underwent surgery presented severe fatigue (53.0% vs 35.5%) (yes vs no); in multivariate analysis, patients who underwent surgery tended to present less severe fatigue compared to patients who did not (aOR, [95%CI] for yes vs no=0.63, [0.40‐1.002]), on the limit of statistically significance. This discrepancy may be explained by the imbalance in those two subgroups as the vast majority of patients underwent surgery (91.4%) with only 8.5% of patients that did not undergo surgery.

This large cross sectional French population‐based study that assessed fatigue and use of health behaviors among cancer survivors, together with previous research suggest the need of global implementation of PA interventions among this population. Nevertheless, we have to acknowledge several limitations. First, fatigue was assessed using the EORTC QLQ C30, which is a validated measure for fatigue among cancer patients, yet does not take into consideration the multidimensional aspects of fatigue. Second, behavioral habits such as PA, were evaluated using self‐reported and nonvalidated questionnaires, lacking granular information, on specific types of activity (eg, aerobic vs resistance training; activities related to working/commuting vs leisure sports activities) and quantity of activity. Third, this study was a cross‐sectional study not allowing a longitudinal evaluation of fatigue and its development and associations over time, making difficult to interpret temporality between fatigue and PA. We cannot exclude that patients with more severe fatigue, might have reduced their PA due to the fatigue. Nevertheless, it allowed us to have an estimate of the problematic of fatigue 2 years after treatment and its relationship with several relevant variables. Fourth, unfortunately many factors that may eventually have a significant impact on fatigue were not available in our dataset and for some variables sample sizes were small, hampering our ability to further and more extensively explore some associations with severe fatigue. Finally, we do not have a healthy comparison population to be able to assess the prevalence and correlates of fatigue in a matched cohort.

## 5 CONCLUSION

In a French population‐based cohort of cancer survivors, we found that fatigue is a substantial problem 2 years after diagnosis. Although cancer patients report high levels of pre‐diagnosis PA, we found an important decrease in PA posttreatment that is significantly associated with risk of severe fatigue. This study raises further awareness regarding the need of applying targeted interventions in this context to improve the health and well‐being of cancer survivors.

## CONFLICT OF INTEREST

Ines Vaz‐Luis‐ Honoraria: Novartis, Astra‐Zeneca, Kephren and the other authors do not have any conflict of interest regarding this article.
