# Supplementary material for: Fatigue and physical activity in cancer survivors: A cross‐sectional population‐based study
Source: Cancer Med. 2019 Mar 12;8(5):2535–44. doi: 10.1002/cam4.2060 (PMC6536944; doi:10.1002/cam4.2060)
Supplement: Supplementary file 1 [file CAM4-8-2535-s001.docx]

**Supplementary table 1:**

| Characteristics | Whole cohort  N (%) | No severe fatigue  N (%) | Severe fatigue  N (%) |
| --- | --- | --- | --- |
| Breast cancer | | | |
| PA before Dx  Active  Inactive  Missing | 1056 (87.1)  153 (12.6)  4 (0.3) | 397 (37.6)  53 (34.6)  2 (50.0) | 659 (62.4)  100 (65.4)  2 (50.0) |
| Change in PA since Dx  Increased/maintained PA  Decreased PA/remained inactive  Missing | 557 (45.9)  651 (53.7)  5 (0.4) | 274 (49.2)  176 (27.0)  2 (40) | 283 (50.8)  475 (73.0)  3 (60) |
| Prostate Cancer | | | |
| PA before Dx  Active  Inactive  Missing | 387 (90.9)  35 (8.2)  4 (0.9) | 287 (74.2)  21 (60.0)  4 (100) | 100 (25.8)  14 (40.0)  0 (0) |
| Change in PA since Dx  Increased/maintained PA  Decreased PA/remained inactive  Missing | 219 (51.4)  202 (47.2)  5 (1.2) | 185 (84.5)  122 (60.4)  5 (100) | 34 (15.5)  80 (39.6)  0 (0) |
| ColoRectal cancer | | | |
| PA before Dx  Active  Inactive  Missing | 297 (86.1)  43 (12.5)  5 (1.5) | 165 (55.6)  28 (65.1)  5 (100) | 132 (44.4)  15 (34.9)  0 (0) |
| Change in PA since Dx  Increased/maintained PA  Decreased PA/remained inactive  Missing | 135 (39.1)  205 (59.4)  5 (1.5) | 98 (72.6)  95 (46.3)  5 (100) | 37 (27.4)  110 (53.7)  0 (0) |
